# A Target for Intervention: Poor Adherence to Follow-Up After Sleeve Gastrectomy in Adolescents and Young Adults

**DOI:** 10.1007/s11695-025-07767-y

**Published:** 2025-03-01

**Authors:** Curry Sherard, Allison B. Frederick, Aaron Lesher, Mary Kate Bryant

**Affiliations:** 1 Medical University of South Carolina, Charleston, SC USA; 2 Ralph H. Johnson VA Medical Center, Charleston, SC USA

**Keywords:** Bariatric surgery, Sleeve gastrectomy, Adolescent bariatric surgery, Obesity

## Abstract

**Background:**

Nonadherence to follow-up after bariatric surgery is associated with lower long-term weight loss. Yet limited data exists on the youngest bariatric population, adolescents and young adults (AYA), who experience life changes in social, psychological, and behavioral domains that can interrupt follow-up. To better understand how age groups affected health outcomes in these populations, this study compared bariatric clinic follow-up adherence between AYA and assessed the impact of follow-up interruption on weight loss.

**Methods:**

Using an institutional registry, we retrospectively reviewed adolescents (age 14–18) and young adults (YA) (age ≥19–26) who underwent sleeve gastrectomy between January 2018 and May 2023. Primary outcome was follow-up compliance (1, 3, 6, 12, 18, 24 months). Secondary outcomes included median total weight loss percentage (%TWL). Lost to follow-up (LTF) was determined by the last bariatric clinic visit attended.

**Results:**

Of 73 (46.8%) adolescents and 83 (53.2%) YA, median preoperative BMI was higher in adolescents (51.0 [44.5,56.8] vs. 48.5 [43.4,51.7], *p* = 0.015). Median total weight loss percentage (%TWL) was greater in YA up to 6 months postoperatively (23.3 [20.5,27.4] vs. 20.2 [15.1,24.9], *p* = 0.008) but did not differ afterward. Median missed follow-up appointments were similar between adolescents (3[3,4]) and YA (4[3,4]). Adolescents were more likely to be LTF at 6 months (34.3% vs. 20.5%, *p* = 0.053). Patients in both age groups were more likely to be LTF if %TWL was in the lowest tertile at 6 months (OR 4.78, 95% CI [2.04, 11.18], *p* =  < 0.001) or 1 year (OR 18.45, 95% CI [5.75, 59.2], *p* < 0.001).

**Conclusions:**

Clinic adherence in the post-bariatric AYA population is poor especially among patients with less %TWL. Identifying when AYA are at risk of LTF allows for targeted interventions to maximize adherence and potentially improve long-term health.

## Introduction

The prevalence of obesity is steadily increasing worldwide, and younger populations are particularly affected [1–7]. In 2020, an estimated 60 million children were classified as obese across the globe [1]. In the USA specifically, more than one-third of children and adolescents are either overweight or obese [2]. Rates of severe obesity have increased more quickly than those of non-severe obesity with 5–7% of children in the USA suffering from severe obesity in 2016 [1, 3, 4]. Childhood obesity continues into adulthood, as children with obesity have a 77% chance of suffering from adulthood obesity as compared to a 7% chance in non-obese children [1, 5]. In turn, more adolescents are turning to bariatric surgery before the age of 21 [5]. Obesity increases the risk for many comorbidities including cardiovascular disease, cancer, type 2 diabetes mellitus (T2DM), osteoarthritis, chronic kidney disease, hypertension, dyslipidemia, obstructive sleep apnea, nonalcoholic steatohepatitis, and psychosocial distress that impairs quality of life [3, 5–8]. The risk of development of these comorbidities is increased in adolescents due to the cumulative effect of long-term obesity and is associated with reduced life expectancy for this age group [3, 5, 6, 9].

Conservative treatments including diet modification, exercise, and medical management have traditionally failed to prevent the long-term consequences of obesity, leading to the evolution of bariatric surgery as the treatment of choice for refractory obesity [1, 3, 5–11]. Bariatric surgery has been shown to be safe and efficacious in both adolescents and young adults (AYA) and is now the standard of care to achieve weight loss and resolution of comorbidities [5–11]. However, adherence to follow-up is crucial to achieving long-term weight loss and maintaining overall health, and younger age is associated with an increased risk of being lost to follow-up (LTF) after bariatric surgery even when not specifically studied in AYA [4, 12–21]. Increasing awareness of the developmental changes that occur during adolescence and young adulthood has led clinicians to focus on the specific needs of these populations, particularly in oncology [22, 23]. The AYA population is unique in that early intervention with bariatric surgery has the potential to benefit them for more years, but they experience many life disruptions in numerous domains during this time that interrupt follow-up. This study specifically sought to compare the clinic follow-up adherence of AYA after bariatric surgery, assess the impact of clinic follow-up interruption on weight loss, evaluate the relationship between weight loss and follow-up adherence, and identify time points for targeted interventions to prevent loss to follow-up in AYA populations.

## Materials and Methods

### Study Design and Groups

Patients aged 14–26 who underwent bariatric surgery between January 2018 and May 2023 at a single institution were included in this retrospective cohort study. All included patients underwent laparoscopic or robotic-assisted sleeve gastrectomy. Patients who underwent Roux-en-Y gastric bypass, duodenal switch, single anastomosis duodenal switch, or revisional operations were excluded.

An institutional registry was used to identify patients 14–26 years old who underwent sleeve gastrectomy during the study period. Patients aged 14 to 18 years were classified as adolescents, and patients aged ≥19–26 years were classified as young adults as defined by the Department of Health and Human Services. Although there is no formal consensus on the definition of young adulthood, we chose to use the narrower range defined by the Society for Adolescent Medicine and the National Academy of the Sciences [24]. This study was deemed exempt from review by the Institutional Review Board at the study center.

### Data Collection and Statistical Analysis

Study data were obtained from the electronic medical record (EMR) and combined with data from the prospectively collected institutional Metabolic and Bariatric Surgery Accreditation and Quality Improvement Program (MBSAQIP) database. The MBSAQIP database is a joint venture with the American College of Surgeons and the American Society for Metabolic and Bariatric Surgery which prospectively tracks 30-day outcomes in a de-identified national database from MBSAQIP centers [25]. While the MBSAQIP tracks many data points, more long-term data including weight data was abstracted from the EMR, including weight recorded at all follow-up visits and missed appointment information. Post-operative visits were standardized in our bariatric program at 1, 3, 6, 9, 12, 18, and 24 months postoperatively followed by annual visits. Our institution has separate pathways prior to surgery for adolescents and adults. However, the number of touchpoints after surgery is equivalent for both age groups. The most notable difference in the pathways is that many adolescents are enrolled in a longer nutritional program prior to consideration of bariatric surgery. All adult patients undergo social work evaluations preoperatively, and patients who are identified as needing further support undergo behavioral medicine evaluations. All adolescents undergo rigorous behavioral medicine evaluation by specialized providers in accordance with ASMBS guidelines. These evaluations aim to identify social support and individual readiness to undergo surgery. Social work and behavioral medicine appointments are continued postoperatively on an individualized basis if needs are determined via screening at the predetermined postoperative time points. Nicotine and alcohol use is assessed preoperatively in all patients, and the institution has a strict 90-day preoperative nicotine abstinence policy. As such, none of the patients was actively using nicotine during the perioperative period. Mental health, social work, or dietician appointments addressing other medical or social aspects of a patient’s care outside of weight-associated issues were not included specifically in this study.

### Clinical Outcomes

The primary outcomes were adherence to clinic follow-up and median total weight loss percentage (%TWL) at the specified time periods overall and stratified by age subgroup. LTF was defined as not returning after a specific post-operative time point to any bariatric-related visit and was measured cumulatively. Visits delayed up to 30 days after a particular time point were included in the data for that time point. Descriptive statistics for patient characteristics and outcomes were reported as percentages, mean ± standard deviation (SD), or median with interquartile range (IQR), as appropriate for the distribution. Where appropriate, differences between groups were examined by Fisher’s exact test, chi-squared test, or Kruskal–Wallis test. Univariate logistic regression analysis was used to compare weight loss and adherence to follow-up among the two groups. Regression results are reported as odd ratios with 95% confidence intervals (CI). Statistical significance was defined as *p*-value ≤ 0.05. Missing data were excluded from calculations that were specific to that field. As missingness was an outcome, this is reported for each time point of follow-up, where applicable. All analyses were performed using Stata v16 (StataCorp 2021, College Station, TX, USA).

## Results

### Overall Patient Cohort

The final study cohort (*n* = 156) included 73 adolescents (46.8%) and 83 young adults (53.2%) over the 5-year period of surgery. Adolescents were more likely to be male than young adults (34.2% vs. 8.4%, *p* < 0.001) and had a higher body mass index (BMI) before surgery (median 51.0 [44.5, 56.8] vs. median 48.5 [43.4, 51.7]). Adolescents more frequently had obstructive sleep apnea requiring continuous positive airway pressure (19.2% vs. 10.8%, *p* = ns), and young adults more frequently had hypertension treated with medication (14.5% vs. 6.9%, *p* = ns). Demographic information and comorbidities of the study population are included in Table 1.

**Table 1:** Demographic information and comorbidity profile of adolescents and young adults undergoing bariatric surgery

Variable	Overall cohort *N* = 156 *n* (%)	Adolescents *N* = 73 *n* (%)	Young adults *N* = 83 *n* (%)	*p*-value
Age, median (IQR)	19.8 (17.3, 24.2)	17.0 (15.8, 18.0)	24.1 (21.6, 25.7)	< 0.001
Weight closest to surgery (kg), median (IQR)	139.2 (121.6, 159.3)	147.9 (127.6, 169.7)	134.7 (120, 148.8)	0.002
BMI closest to surgery, median (IQR)	49.4 (43.7, 54.5) Range 31.8–82.8	51.0 (44.5, 56.8)	48.5 (43.4, 51.7)	0.015
Female gender	124 (79.4)	48 (65.8)	76 (91.6)	< 0.001
Race				0.085
White	66 (42.3)	30 (41.1)	36 (43.4)	
Black	85 (54.5)	43 (58.9)	42 (50.6)	
Unknown	5 (3.2)	0	5 (6.0)	
Hispanic ethnicity	7 (4.5)	0	7 (8.4)	0.011
Payor status, *N* = 246				0.109
Medicaid	82 (69.5)	39 (81.3)	43 (61.4)	
Medicare	2 (1.7)	0	2 (2.9)	
Other	5 (4.2)	2 (4.2)	3 (4.3)	
Private	25 (21.2)	7 (14.6)	18 (25.7)	
Self-pay	4 (3.4)	0	4 (5.7)	
Comorbid conditions*				
OSA requiring CPAP	23 (14.7)	14 (19.2)	9 (10.8)	0.143
GERD	15 (9.6)	10 (13.7)	5 (6.0)	0.105
Insulin-dependent diabetes	5 (3.2)	4 (5.5)	1 (1.2)	0.130
Noninsulin-dependent diabetes	18 (11.5)	11 (15.1)	7 (8.4)	0.196
Median preoperative HgA1C	5.3 (5.1, 5.6)	5.4 (5.2, 5.7)	5.2 (5.0, 5.5)	0.049
Hypertension requiring medication	17 (10.9)	5 (6.9)	12 (14.5)	0.128
Number of patients on 2 or more anti-hypertension medications	8 (5.1)	1 (1.4)	7 (8.4)	0.098

*Comorbid conditions with less than 5% incidence: severe COPD (0.6%), history of PE (0.6%), prior cardiac surgery (1.9%), immunosuppressive therapy (3.2%), anticoagulation (1.9%), ESRD on dialysis (0.6%)

### Postoperative Follow-Up

**Fig. 1:**
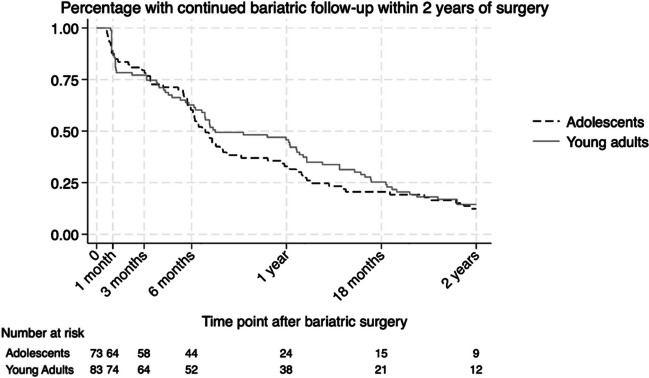
Incidence of nonadherence and loss to follow-up of adolescents and young adults after bariatric surgery

Adolescents were more likely than young adults to be LTF after the 6-month postoperative visit (34.3% vs. 20.5%, *p* = 0.053). Rates of LTF between groups did not differ at other time points. At 12 months postoperatively, 75.3% of adolescents and 72.3% of young adults were LTF (*p* = ns). This cohort grew to include 93.1% of adolescents and 87.9% of young adults LTF at 24 months postoperatively (*p* = ns). Overall follow-up data for AYA is shown in Fig. 1. The percentage of AYA LTF at the specified postoperative timepoints is shown in Table 2.

**Table 2:** Percentage of adolescents and young adults lost to follow-up at specified postoperative time points

	Adolescents *N* = 73 *n* (%)	Young adults *N* = 83 *n* (%)
Lost at 30 days	12 (16.4)	19 (22.9)
Lost at 3 months	20 (27.3)	25 (30.1)
Lost at 6 months	45 (61.6)	42 (50.6)
Lost at 12 months	55 (75.3)	60 (72.3)
Lost at 18 months	61 (83.5)	70 (84.4)
Lost at 2 years	68 (93.1)	73 (87.9)

### Weight Loss Outcomes

**Fig. 2:**
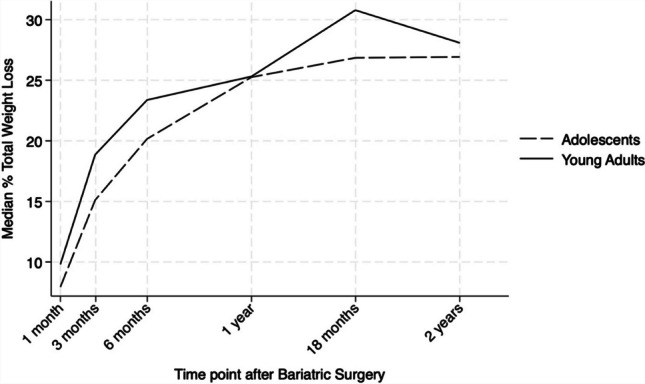
Median total weight loss percentage (%TWL) after bariatric surgery at postoperative time points, stratified by age group

Median %TWL was higher among young adults at 1 month (9.8 [7.9,10.6] vs 7.9 [6.2,9.4], *p* = 0.002), 3 months (18.9 [16.6,21.7] vs 15.1 [11.9,18.3], *p* = 0.002), and 6 months (23.3 [20.5,27.4] vs. 20.2 [15.1,24.9], *p* = 0.008). At 1-year follow-up and afterwards, median %TWL was similar between age groups, *p* = ns (Fig. 2). Overall %TWL among AYA is shown in Table 3.

**Table 3:** Median total weight loss percentage (%TWL) in the overall cohort, adolescents, and young adults after bariatric surgery at postoperative time points

Median (IQR) %TWL	Overall cohort *N* = 156	Adolescents *N* = 73	Young adults *N* = 83	*p*-value
30 days	8.7 (6.9, 10.3)	7.9 (6.2, 9.4)	9.8 (7.9, 10.6)	0.002
3 months	16.7 (13.9, 19.8)	15.1 (11.9, 18.3)	18.9 (16.6, 21.7)	0.002
6 months	21.9 (18.7, 26.7)	20.2 (15.1, 24.9)	23.3 (20.5, 27.4)	0.008
1 year	25.3 (21.2, 29.9)	25.3 (20.7, 29.6)	25.3 (21.2, 32.4)	0.984
18 months	26.9 (19.2, 35.5)	26.9 (15.4, 29.4)	30.8 (19.5, 37.3)	0.262
2 years	28.1 (23.2, 30.6)	26.9 (23.5, 32.3)	28.1 (21.4, 30.4)	0.817

To determine the relationship between %TWL and maintaining engagement with our bariatric program, we stratified patients into tertiles based on their %TWL. Patients in both age groups were more likely to be LTF if %TWL was in the lowest tertile at 6-month visit (OR 4.78, 95% CI [2.04, 11.18], *p* =  < 0.001) or at 1-year visit (OR 18.45, 95% CI [5.75, 59.2], *p* < 0.001).

The overall cohort was stratified by Black and White race to determine if %TWL or follow-up differed by race. Subgroup analysis by race was limited to these two groups and did not include Hispanic ethnicity given the small sample (< 5%) in our cohort. Follow-up visit adherence was similar at all time points between Black and White patients, *p* = ns. While median BMI at baseline was comparable between Black patients (BMI 49.6 [43.9,55.10]) and White patients (49.3 [43.0,54.1]), Black patients had lower %TWL than White patients at 6 months (%TWL 20.1% vs 24.0%, *p* = 0.004) and 12 months (%TWL 23.7% vs 27.5%, *p* = 0.05) postoperative. Black patients were significantly more likely to be LTF after the 6-month postoperative visit if their %TWL was in the lowest tertile (OR 10.12, 95% CI [3.11, 32.94], *p* < *p* < 0.001) while this effect was not significant for White patients (OR 2.22, 95% CI [0.49, 9.89], *p* = 0.295). Overall %TWL stratified by race is shown in Table 4.

**Table 4:** Median total weight loss percentage (%TWL) in combined population of adolescents and young adults after bariatric surgery at postoperative time points, stratified by race.*

Median (IQR) %TWL	Black patients *N* = 85	White patients *N* = 66	*p*-value
30 days	8.7 (6.7, 10.4)	9.2 (7.2, 10.3)	0.504
3 months	16.8 (13.1, 20.1)	16.7 (13.9, 19.2)	0.912
6 months	20.1 (16.5, 25.7)	24.0 (20.5, 27.4)	0.004
1 year	23.7 (17.3, 28.6)	27.5 (22.6, 32.9)	0.054
18 months	26.9 (16.9, 34.7)	28.1 (22.6, 43.2)	0.329
2 years	26.9 (21.4, 30.6)	29.9 (26.9, 41.9)	0.296

^*^Excludes 5 patients with unknown race

## Discussion

In this study, we sought to understand the relationship of bariatric clinic follow-up to weight loss in a population of AYA who, historically, are at a higher risk of poor clinic follow-up compared to older patients [15, 20, 21]. In the literature, greater adherence to follow-up has consistently shown to be beneficial to patients through higher post-operative weight loss, the avoidance of weight regain, and fewer post-operative complications in patients who come to clinic as prescribed [4, 12, 13, 15, 17, 18, 20, 21]. Adherence to follow-up is associated with an increased percentage of excess body weight loss (%EBWL) and is necessary for improvement of obesity-related comorbidities [4, 12–18]. Furthermore, long-term adherence to care is associated with fewer rehospitalizations, more positive lifestyle behaviors, and increased use of vitamin supplementation postoperatively [19]. Finally, adherence to follow-up is necessary for early detection and treatment of adverse outcomes, complications, and nutritional deficiencies [12, 13, 20]. Risk factors for non-adherence to follow-up include %EBWL < 50% and young age [21].

The unique nature of the AYA population in the healthcare setting has been best investigated in AYA cancer patients. In a review article by Rosenthal et al. examining the general AYA oncology population, AYA cancer patients are less likely to be adherent to follow-up and more likely to experience inferior clinical outcomes than their older counterparts [22]. The factors shown in this study to impact outcomes including financial strain due to health care interventions, changes in insurance from a parental plan to an individual plan, and disruption in ability to work are also applicable to AYA bariatric patients [22, 24]. Other challenges identified in the AYA population included leaves of absence from school, perceived isolation in a developmental phase during which peer relationships are highly valued, and hesitancy to seek help due to a variety of psychosocial barriers [22]. Because of their unique concerns, interventions must be tailored to the AYA population to be effective. While cancer patients and bariatric patients have important differences including long-term survival and treatment toxicity, many of the barriers to care identified in these studies exist because of the unique developmental stages that AYA patients experience and can apply to AYA patients in general. Therefore, these studies highlight the financial, interpersonal, and professional barriers to care that exist in AYA cancer patients that may also impact AYA bariatric patients. Targeting interventions to address this young population and its specific challenges may be effective in bariatric patients as well.

Our study demonstrates an extremely low rate of clinic follow-up in these younger populations during the study period. This is understudied in the literature with few studies addressing this problem. In a study by Inge et al., follow-up adherence in adolescents was up to 90% at 1 year after Roux-en-Y gastric bypass surgery [11]. However, this study was done under the auspices of an NIH-sponsored trial with strict metrics. In contrast, our current study found that approximately 75% of adolescents and 72% of young adults were lost at 1-year follow-up in the setting of a high-volume, quaternary care academic medical center. The longitudinal bariatric surgery follow-up clinic at the study institution has a standard pathway for both adolescents and adults, with routine virtual reminders of visits through text messaging and the EMR messaging system. Additionally, this program is the only adolescent bariatric program in the state, and patients often travel long distances for care. A comprehensive virtual visit program was instituted in 2020 during the COVID-19 pandemic, and remained in place, but LTF rates have continued to be low during this period as well. While we did not study older populations, historical data suggests that follow-up rates are much higher in pooled adult populations [21].

Regarding potential timing of intervention, the 6-month postoperative visit appears to be a crucial time point for adolescents as LTF is high risk after this visit. The relative retention of young adults versus the loss of adolescents is potentially explained by the greater %TWL young adults experience until this time. This could be reflective of reduced adherence among adolescents to dietary and exercise recommendations, but it also could result in decreased motivation among adolescents to continue adhering to recommendations after this period. The observation that patients in both age groups with less %TWL were more likely to be LTF at 6 months and 1 year provides further evidence that the few months following surgery is a crucial time for intervention and suggests that patients with less %TWL at early follow-up visits should be identified as being at risk. Targeting AYA with aggressive intervention early in their postoperative course could improve %TWL and increase retention at later visits.

The impact of these findings could lead to interventions to improve weight loss outcomes in these vulnerable populations. Younger patients are increasingly afflicted by obesity-related comorbidities that were historically diseases of adulthood [6, 9]. T2DM in particular has become more prevalent in childhood and not only puts younger patients at an increased risk for health consequences due to a longer disease course but also is more aggressive when it occurs in children, with insulin therapy becoming necessary earlier in the disease course for younger patients [3, 6]. While this study focused on sleeve gastrectomy, studies have shown that remission of hypertension and T2DM following gastric bypass occurs more frequently in adolescents than in adults [9]. Thus, AYA may have a greater potential to benefit from bariatric surgery compared to older adults due to resolution of comorbidities.

In addition to age, we sought to further examine the impact of race on the healthcare disparities experienced by these vulnerable populations. The impact of race on bariatric surgery outcomes has been studied with an overall trend suggesting that White patients lose more weight postoperatively than patients belonging to a racial minority group while resolution of comorbidities is similar [26]. This discrepancy in weight loss has not been well demonstrated in younger populations, but few studies have analyzed racial disparities in weight loss in the adolescent bariatric population [27–29]. Our study corroborated previous findings, showing that White patients lost significantly more weight at 6 months and 12 months postoperatively. Additionally, Black patients who had poor weight loss outcomes were significantly more likely to be LTF than White patients with similarly poor weight loss. We were unable to study the impact of ethnicity due to low numbers of Hispanic populations in our study groups.

This study suggests that a light-touch telehealth intervention at 6 months may help improve attendance in the bariatric longitudinal clinic along with reinforcing long-term self-management of healthy lifestyle behaviors after bariatric surgery. Telehealth has been shown to connect patients in many different specialties who may be LTF otherwise [30]. In AYA, telehealth has been shown to increase clinic follow-up adherence by reducing barriers associated with in-person visits [31]. The bariatric population faces the additional burden of multiple clinic appointments with the multidisciplinary team of dieticians, psychologists, social workers, and surgeons. Ideally, these clinic visits are scheduled on the same day, but telehealth can reduce the time spent traveling to clinic appointments especially when they are frequent. As post-bariatric success is dependent on life-long health behaviors, opportunities for counseling and intervention are lost when patients do not follow-up with providers with expertise in the bariatric population. The positive impact of telehealth on clinic no show rates in the preoperative period has also been demonstrated for bariatric patients [32, 33]. Because telehealth has been effective in improving follow-up adherence in the AYA population and in the bariatric population in general, we hypothesize that telehealth may improve postoperative follow-up in AYA bariatric patients. The data presented here will inform endeavors to use a light-touch, individualized approach to post-bariatric care through mobile health modalities.

Limitations of this study include that it was a retrospective cohort study at a single center and therefore cannot capture national trends in follow-up and long-term outcomes among AYA. Because the end-date of the study period was within 1-year of data analysis, long-term outcomes could not be studied in patients undergoing surgery towards the end of the study period. Additionally, the exclusion of patients who received revisional operations could exclude patients with worse outcomes and less %TWL, although only 4 patients in this dataset underwent revisional surgery. Future studies should further examine socioeconomic factors, mental health, and other preoperative risk factors such as risk behaviors that contribute to loss to follow up and lower weight loss to more effectively target at-risk individuals and tailor interventions accordingly. Due to the deidentified nature of this study, we were not able to analyze how psychosocial factors such as family support impacts follow-up or weight loss. Finally, even though weight loss was similar among AYA in this study, weight loss data is limited to those patients who adhere to follow-up. Therefore, it is possible that results could change if the current weights of all patients, regardless of follow-up status, were known. Current efforts are underway to capture post-operative weights from non-bariatric clinic or ER visits using EMR data from outside healthcare systems.

In summary, AYA are more likely to be LTF after bariatric surgery than their older counterparts with rates as high as 75% at 1 year. AYA who lose less weight are at higher risk for LTF, and this effect is amplified among Black patients. This is understudied in the literature, and the AYA population is particularly vulnerable to adverse long-term health outcomes that are associated with lack of follow-up. The adoption of technology among patients in this age group provides a unique opportunity for intervention as these patients are more easily reached through virtual modalities than older generations. The use of technology coupled with the identification of vulnerable time points at which many patients are LTF allows for the development of targeted, multidisciplinary interventions within the first year postoperatively to prevent follow-up nonadherence and thereby improve long-term patient outcomes.

## Data Availability

No datasets were generated or analysed during the current study.
